# Unregulated provider perceptions of audit and feedback reports in long-term care: cross-sectional survey findings from a quality improvement intervention

**DOI:** 10.1186/1471-2318-13-15

**Published:** 2013-02-13

**Authors:** Kimberly D Fraser, Hannah M O’Rourke, Melba Andrea B Baylon, Anne-Marie Boström, Anne E Sales

**Affiliations:** 1 Faculty of Nursing, University of Alberta, Edmonton, AB, Canada; 2 Department of Neurobiology, Care Science and Society, Division of Nursing, Karolinska Institutet, Huddinge, Sweden; 3 Department of Geriatric Medicine, Danderyd Hospital, Danderyd, Sweden; 4 School of Nursing, University of Michigan, Ann Arbor, MI, USA

**Keywords:** Long-term care, Unregulated care provider, Quality of care, Audit and feedback

## Abstract

**Background:**

*Audit with feedback* is a moderately effective approach for improving professional practice in other health care settings. Although unregulated caregivers give the majority of direct care in long-term care settings, little is known about how they understand and perceive feedback reports because unregulated providers have not been directly targeted to receive audit with feedback in quality improvement interventions in long-term care. The purpose of this paper is to describe unregulated care providers’ perceptions of usefulness of a feedback report in four Canadian long-term care facilities.

**Methods:**

We delivered monthly feedback reports to unregulated care providers for 13 months in 2009–2010. The feedback reports described a unit’s performance in relation to falls, depression, and pain as compared to eight other units in the study. Follow-up surveys captured participant perceptions of the feedback report. We conducted descriptive analyses of the variables related to participant perceptions and multivariable logistic regression to assess the association between perceived usefulness of the feedback report and a set of independent variables.

**Results:**

The vast majority (80%) of unregulated care providers (n = 171) who responded said they understood the reports. Those who discussed the report with others and were interested in other forms of data were more likely to find the feedback report useful for making changes in resident care.

**Conclusions:**

This work suggests that unregulated care providers can understand and feel positively about using audit with feedback reports to make changes to resident care. Further research should explore ways to promote fuller engagement of unregulated care providers in decision-making to improve quality of care in long-term care settings.

## Background

Internationally, there has been increased emphasis on improving the quality of care in continuing care settings (that is, home care, supportive living, and facility-based long-term care) [1-3]. There has also been an emphasis on unregulated care providers’ role in delivering high quality care in long-term care (LTC) settings [4,5]. *Audit with feedback* is a quality improvement approach that is moderately effective for improving regulated professional practice in several health care settings [6,7]. However, little is known about how unregulated care providers understand and perceive feedback reports in LTC settings. The purpose of this paper is to explore the perception of audit with feedback reports by unregulated providers in LTC settings.

### The long-term care (LTC) setting

Residents within facility-based LTC settings in Canada are vulnerable people who need substantial personal care and on-site, 24-hour nursing care [1,8,9]. “Personal care” refers to help with daily living activities (e.g. bathing and eating) and other therapeutic interventions (e.g. medication reminders), as well as managing behaviour related to diseases (e.g. dementia-induced wandering) [3].

A current trend in caring for older Canadians experiencing functional or cognitive decline (or both) is to provide supportive care within a person’s home or in an assisted living setting for as long as possible [9]. This “aging in place” approach (8), along with supporting individuals and families for as long as possible within community settings, is emphasized in numerous Canadian jurisdictions [1,9], including Alberta [10]. However, many individuals or their families still choose LTC settings, often when community support can no longer manage the patient’s substantial health needs [8,9]. In Alberta in 2008, 14,500 seniors or persons with disabilities (or 0.40% of the province’s population), lived in a LTC facility [10,11].

In 2010, there were 2136 Canadian LTC facilities with 212,948 staffed beds. In Alberta in 2010, there were 199 LTC facilities with 18,738 staffed beds [12]. While Canadian LTC residents often require less medical, nursing, or personal care compared to hospital patients [1], they tend to have higher levels of dependency and cognitive impairment than seniors living in the community [9]. In Alberta specifically, LTC residents’ care needs were 35% higher in 2003 than in 1990. With 75% of these residents in the highest of three categories of need—specifically for functional care [8] —these data suggest an increase in care intensity over time.

### Unregulated care providers

LTC in Canada is a provincial responsibility [13] and, as a result, characteristics of LTC environments vary from province to province [1]. However, common across all Canadian LTC settings is the high proportion of unregulated care providers (that is, unlicensed staff who may also be referred to as care aides, nurses aides, or personal care workers in other jurisdictions across Canada and abroad) who deliver the majority of front-line, direct care to residents [14]. In 2007, 72% of care providers in LTC in Alberta were unregulated caregivers; regulated professionals (like registered nurses (RNs)/registered psychiatric nurses (RPNs) and licensed practical nurses (LPNs)) comprised only 17% and 11%, respectively [15].

Unregulated caregivers give basic care, including personal and supportive care, under the supervision of a regulated professional (an RN/RPN or LPN) [3,16,17]. They also recognize and report resident symptoms that require a regulated health professional’s intervention [8]. Hence, they have been described as the “backbone” of LTC [16,18].

Educational preparation of unregulated providers varies widely, especially given a high proportion are educated immigrants who do not have professional licensure in Canada. In comparison with RNs and LPNs who have either a mandatory four-year baccalaureate degree or a 15-month to two-year diploma [8,19], unregulated caregivers generally have lower levels of education. Many unregulated caregivers train on the job; some have obtained a personal care attendant certificate by attending a 12 to 40 week program delivered through a college or vocational school [8]. Alberta, along with many other Canadian provinces, has no mandatory or standardized approach for educating unregulated care providers [8] and the Government of Alberta recognizes the need to certify its approximately 16,000 unregulated providers, as one approach to ensure provision of quality care [4]. The province developed competency profiles [20] and, since 2008 has been considering whether all unregulated care providers will be required to obtain a recognized education certificate or complete a competency assessment.

### Quality of care

The increasing complexity and intensity of care needs in LTC residents result in “challenges in meeting human resources and continuing staff education needs” [8], p. 22]. As well as increasing numbers of LTC residents, a wide range of challenging co-morbidities often influence their needs [21], yet it is unregulated care providers (with the lowest level of education and pay) who are most in contact with them [14,16]. LTC thus relies on the least prepared individuals to provide the majority of care to a growing number of older adults with multifaceted health needs [8,22,23]. The high proportion of unregulated care providers may affect the quality of care in LTC settings, as they may be limited in their ability to respond appropriately to residents [14,24].

Broader contextual factors may also impact the delivery of care within LTC settings. Unregulated care providers have little autonomy [14] and decision makers rarely consult them [16,24]. Improving teamwork among the variety of care providers— especially between professional nurses and unregulated care providers—might improve quality of care [25-27]. A partnership approach between unregulated caregivers and management, rather than a hierarchical one, may improve unregulated providers’ quality of care [28]. This approach would empower them, involving them in decision-making [5] and creating a culture in which they are treated with “respect, support, and caring” [5], p. 637]. Thus, including unregulated care providers in activities traditionally left to professionals, such as quality improvement interventions, could be an important way to improve the culture [5,29,30], improving the overall quality of care in LTC settings [31-33].

### Audit with feedback interventions

One approach to improving quality of care is through audit and feedback. This is the auditing of current care practices or resident outcomes and provision of the resulting data as feedback to care providers, in an effort to influence their clinical practice [6,7]. Audit with feedback has the potential to influence health care provider behaviour, because it shows providers how residents in their facility compare to residents in other similar settings in selected areas affected by the care that they deliver [34]. The ultimate goal of an audit with feedback intervention is improving the quality of care [6,7].

Audit with feedback has modest effects on professional practice, with effects tending to be greater in settings with little prior exposure to this type of intervention [6,7,25]. Minimal evidence is available on the effectiveness of audit with feedback in LTC settings and, in particular, the effects when targeting unregulated care providers. In one randomized clinical trial, the researchers targeted the professionals (the LTC administrator and director) with feedback, but not the unregulated care providers. Further, the authors do not report to what extent the professionals passed the information on to other providers in their facility [35]. We currently do not know how unregulated providers, when directly targeted, might perceive and respond to feedback report information within LTC contexts; this study’s results will begin to fill this knowledge gap by identifying how unregulated care providers perceived the information included in the report. Although self-reported intent-to-change behavior is not the focus of this study, unregulated providers’ perceptions of the utility of the feedback report information may be an important initial factor that influences whether a plan to change practice is made. Intent-to-change behavior will be reported in a separate publication.

### The need for formalized audit with feedback processes

In Alberta and several other jurisdictions across Canada and internationally, standardized data are readily accessible for audit with feedback in LTC settings. The data come from the Resident Assessment Instrument-Minimum Data Set version 2.0 (RAI 2.0). The RAI 2.0 is a standardized assessment tool, mandated by Alberta Health and Wellness in LTC settings across Alberta. Developed by the interRAI consortium, it is used within Canada and internationally to assess and document a wide variety of LTC resident characteristics, including physical, mental, and functional status [36,37]. When aggregated to the unit or facility level, RAI 2.0 data also permit estimation of quality indicators. These measure the incidence and prevalence of resident health problems or outcomes that the quality of care within a unit or facility may influence.

Alberta LTC settings have not instituted formalized audit with feedback processes to date. The availability of these standardized data and the lack of a current audit and feedback approach provide an opportunity to test an audit with feedback intervention for care providers and managerial employees in LTC, and to obtain evidence about unregulated caregivers’ perceptions of the feedback report information [38]. The aim of this paper is to describe unregulated caregivers’ perceptions of usefulness of a feedback report within LTC settings in a large urban centre in Alberta, Canada. Specifically, we were interested in understanding whether the reports provide information that unregulated care providers perceive could be useful to provide better quality care to residents, and to what extent other variables were associated with the perception of feedback report utility.

## Methods

### Design and setting

The study protocol (previously published) contains a detailed description of the study design [38]. This paper is a cross-sectional subset of data that examines unregulated provider perceptions of the feedback reports. Monthly feedback reports were prepared and distributed in nine nursing units in four LTC facilities in Edmonton, Alberta, over a 13-month period in 2009–2010. A large public organization owns two of the facilities and a large faith-based non-profit organization owns the others. We conducted post-feedback report surveys with employees from each of the study sites to assess uptake of the audit with feedback intervention. We conducted these surveys one week after distributing the feedback reports, with a few exceptions as described below. The survey assessed respondents’ perceptions of their understanding and their opinion of the usefulness of the monthly feedback report. We used these survey data for our analysis.

We pilot tested both the feedback report and survey instrument before the study. During the pilot, we hand-delivered feedback reports to unregulated providers on each unit and administered a post-feedback report survey one week after the report distribution. We have found that among 85 unregulated care providers who participated in the pilot survey, all of them understood the report but only 61% found the report useful. The methods for report distribution were modified slightly after the pilot to leave some feedback reports and surveys in the break rooms of each of the units, to target any care providers who were either too busy during their shift to participate in the study, or who were not on-site. Minor modifications were made to the survey to improve clarity for several questions.

### Generating and distributing the reports

We based the feedback reports on data received from the RAI 2.0’s most recent quarterly resident assessment data. By aggregating resident data to the unit level, we constructed four quality indicators to include on the feedback report: (i) pain frequency and intensity; (ii) risk of falls; (iii) occurrence of falls; and (iv) depression prevalence. We created line graphs for the feedback reports, each comparing the quality indicators in a given unit to all other units in the study. We used codes to identify units and did not disclose the identity of units. Furthermore, the data from the eight other units was aggregated in the report (i.e. participants compared their unit to the aggregated scores from the eight other units in the study). However, because of the proximity of the units within a facility it is possible that staff may have surmised whether some of the eight other units in the study were from their facility. Explanatory text bullets followed each graph. A sample report and further details of its development is included in the study protocol [38]. Two research assistants (RAs) hand-delivered the reports to available staff in each of the nine LTC units. Additional reports were left in a previously arranged location on the unit. They delivered them during a consistent week in each month for the 13 months of the intervention period.

### Sample and data collection

All staff were offered the opportunity to receive and read the monthly feedback reports and to complete the post-feedback survey. Respondents to the post-feedback survey were a convenience sample of facility administrators, nurse managers, and direct care providers. Direct care providers included unregulated caregivers, RNs, LPNs, physical therapists, recreational therapists, occupational therapists, pharmacists, social workers, and other allied health providers. Each respondent indicated their professional affiliation, where applicable, or occupation on the survey. In this article, we report findings from the unregulated direct care provider survey respondents only.

We measured provider perceptions of the feedback report through a paper-and-pen survey, distributed, in most months, one week after the feedback reports. We did not administer post-feedback report surveys in July and August, or over the holiday season (to avoid low staffing periods and minimize survey fatigue). Thus, there were 9 survey cycles for 13 report distribution periods.

The post-feedback report survey, originally published in the protocol paper [38], had three sections that contained: (i) demographic information about the respondents; (ii) items capturing perceptions of the feedback report; and (iii) items assessing intent to change behaviour (see Additional file 1). This study is focused on the items on perceptions of the feedback report from section ii. RAs visited each unit at different times and on at least two days in the week, and offered all employees the opportunity to complete the anonymous pen and paper survey at their convenience. They directed the respondents to return the surveys to the RA, to the return box at their site, or by mail.

### Analysis

For this paper, we only analysed unregulated care provider responses to the survey. The authors selected potentially relevant variables to explore unregulated providers’ understanding and perceptions of the reports, as there is no existing literature in this area. All variables included in the logistic regression analysis use a dichotomous coding scheme, where 1 indicates a positive response and 0 indicates a negative response. The audit with feedback intervention was targeted to the individual care provider because we wanted to know whether they found the information useful to inform the way that they, personally, delivered care. The survey questions of interest for this analysis are listed below:

1. “How well do you feel you understood the information that was in the report(s) about residents on your unit?”

2. “How useful did you find the report?”

3. “Does getting this feedback report make you more interested in other types of data?” (If yes, an open-ended question followed, asking them to identify which types of information were of interest.)

4. “Did you discuss the report with another staff member in your unit or with someone who works somewhere else in the facility?” (If so, respondents indicated whether they spoke with another staff member to find out what they thought, to obtain advice about how to make things better, or for another reason.)

5. “Did the report give you information that you could use to make changes in the way you take care of residents?”

Variable five was the dependent variable for this analysis. Variable two, which measures general perceptions of usefulness, differs from the dependent variable because a respondent may feel the information is useful in general, but not useful for actually changing how they deliver care.

For this sub-study, we combined data from the last three survey cycles from the 13-month intervention period (with 59, 84, and 112 respondents) for a total of 255 unregulated provider participants. We pooled these surveys to increase statistical power, because of the relatively low numbers of respondents in each survey period. Because providers may or may not take part in all of the surveys, 24 out of the 255 respondents participated in more than one survey during the pooled survey periods, with 21 completing two and three participating in all three. Given that the data were anonymous, we assessed whether respondents had completed more than one survey by linking surveys according to demographic information (that is, the unit they currently work on, the number of years they worked on the unit, and the number of years they worked in a LTC organization). The combined survey data yielded 228 unique respondents from the nine LTC units across the last three survey cycles. We calculated an intra-class correlation coefficient to assess how much information was correlated across different surveys completed by the same respondent. The calculated intra-class correlation coefficient was 0.6, indicative of substantial correlation. Therefore, we only used the data from the first survey completed by a respondent in the analysis.

We also examined inconsistent responses to some variables. A group of respondents reported that they understood half or less than half of the feedback report (variable 1), yet found the report useful (variable 2). We excluded these responses due to the inconsistency between these items. Furthermore, those cases with missing data for any of the study variables were dropped in the multivariable regression analysis.

Categorical and continuous variables from the survey data were analyzed descriptively by calculating proportions and means, respectively. We analyzed the open-ended question (on what additional information would be interesting) using content analysis. For bivariate analysis, we used a chi-square test to test the association between each of the variables selected and the dependent variable. For the multivariable logistic regression, we included all variables tested in the bivariate analyses, regardless of their level of significance in the bivariate analysis. Multivariable logistic regression analyses included a cluster correction to adjust for the effect of the nursing unit (using the cluster command in Stata version 10.0). We carried out all statistical tests at the 5% significance level.

### Ethical considerations

The Human Research Ethics Board at the University of Alberta approved this study. All participating LTC sites gave us operational approval. Informed consent was obtained from all participants.

## Results

We obtained completed surveys with valid responses to the study variables for 171 unregulated providers for inclusion in the analyses. Unregulated care providers had worked an average of ten years within LTC settings (s.d. 8.58; range 0 to 35) and an average of five years within their current long-term care unit (s.d. 5.01; range 0 to 22). In general, the majority of participants reported that they found the reports understandable (80%), useful in general (68%), and useful for making changes to resident care (69%) (Table 1). Of the 88 respondents who were interested in other data, 46 indicated one or more types of data that were of interest to them, the most common of which (26%) were improvement strategies. Other data of interest included information about the nursing unit (20%), patient behaviour and wellbeing (17%), physical health concerns (17%), quality indicator details (11%), and others (13%). Those who discussed the report with another staff member had two main reasons for doing so: (i) wanting to find out what other employees thought of the report (49%), and (ii) obtaining advice from other care providers on how to improve resident care based on the report (57%).

**Table 1:** Proportion of unregulated care providers with a positive response to each variable (n = 171)

**Study variable**	**n**	**%**
Report gave useful information to make changes in resident care	118	69.00
Understand more than half of the report	137	80.12
Usefulness of the report	117	68.42
Discussing the report with other staff	71	41.52
Interest in other data	88	51.46

### Associations with usefulness for resident care

The bivariate analysis for each of the independent variables with the dependent variable revealed two significant associations (Table 2). The variables “reporting interest in other types of data” (p = 0.001) and “discussing the report with another employee” (p = 0.007) were significantly associated with the dependent variable (report provides useful information to change care). Perceptions of “usefulness of the report” (p = 0.061) and “understanding more than half the report” (p = 0.545) were not associated with the dependent variable.

**Table 2:** Bivariate associations (n = 171) with ‘report gave useful information to make changes in resident care’

**Independent variable**	**No**	**Yes**	**p-value**
Understand more than half of the report	41 (29.93%)	96 (70.07%)	0.545
Usefulness of the report	31 (26.50%)	86 (73.50%)	0.061
Discussing the report with another staff	14 (19.72%)	57 (80.28%)	0.007
Interest in other data	17 (19.32%)	71 (80.68%)	0.001

*Note:* p-value based on chi-squared test with alpha set at 0.05.

Of the four variables included in the logistic regression model (Table 3), three were statistically significant: (i) assessing the report as useful, in general (OR 2.70, 95% CI 1.19 to 6.16); (ii) discussing the report with another employee (OR 2.15, 95% CI 1.09 to 4.23); and (iii) reporting interest in other types of data (OR 2.67, 95% CI 1.29 to 5.51). Understanding more than half of the report was not associated with the perception that the report gave useful information for changing resident care.

**Table 3:** Multivariable logistic regression (n = 171) with ‘report gave useful information to make changes in resident care

**Independent variable**	**OR**	**SE**	**z**	**p-value**	**95% CI**
Understand more than half of the report	0.47	0.26	−1.36	0.17	0.16 to 1.39
Usefulness of the report	2.70	1.14	2.36	0.02	1.19 to 6.16
Discussing the report with other staff	2.15	0.74	2.21	0.03	1.09 to 4.23
Interest in other data	2.67	0.99	2.66	0.01	1.29 to 5.51

## Discussion

We found that unregulated providers in LTC settings express both an ability to understand feedback reports presented as part of a quality improvement intervention, and perceive that these reports provide them with information that is useful for changing resident care. Prior studies often excluded unregulated providers from quality improvement interventions (such as the distribution of feedback reports), sometimes because the researchers believed these providers cannot understand or use the reports in a meaningful way. The existing literature highlights the lack of education and concerns about unregulated providers’ capacity to understand potentially complex information that may impact quality of care, such as that included in our feedback report.

We believe our study represents the first attempt to deliver feedback reports of this type to unregulated care providers, and the first to report on their level of understanding. The findings of our study suggest that excluding unregulated care providers from quality improvement interventions may be inappropriate, especially given the large proportion of unregulated providers giving care in LTC settings [15]. Our results suggest unregulated care providers should be explicitly included in quality improvement efforts such as feedback reports, rather than excluded, as has often been the case in prior studies.

We anticipated a positive relationship between perceptions of understanding more than half of the report and thinking the report gave useful information for changing resident care (i.e. that a higher degree of understanding would correlate with more positive perceptions of usefulness). However, self-reported understanding of more than half of the report was not related to finding the report more useful for changing patient care.

One possible explanation for this finding relates back to the limited decision-making power that unregulated care providers have within these LTC settings. Our audit with feedback intervention evaluated the effects of the feedback reports on individuals. However, unregulated care providers work within complex organizations, often operating in hierarchical management structures. This leaves the unregulated care provider with little opportunity to make independent decisions about resident care [14,39]. Thus the degree to which the unregulated care provider understands the feedback report (i.e. understanding more than half of the report versus less than half) may be much less important to the ability of the unregulated provider to make changes to care than are those contextual elements of the unregulated provider’s work environment that may make it challenging to use the information.

Ratings of usefulness are likely to have been affected by whether unregulated care providers perceived the report as useful for their own personal practice, or in collaboration with others. Findings from the logistic regression analysis support this, indicating that those who discussed the report with others were more likely to rate the report as providing information that was useful in making changes to resident care. This positive association suggests collaborating may be important to using information in resident care. Validation by peers and other care provider groups may be a mechanism for empowerment. In a related note, while the positive association in the multivariable logistic regression between general perceptions of usefulness and considering the report useful to make changes in resident care seems relatively unremarkable, it is interesting that these two variables are not more highly associated, and are not significantly associated in the bivariate analysis. That is, providers may find the report useful, in general; however, this may not be associated with perceiving that the report provides information that is useful for making changes to resident care until after adjustment for other variables including, “discussing the report with others”.

Finally, we observed an association between the expression of interest in additional data with the variable measuring perceived usefulness of the feedback report for changing resident care. This suggests that the receipt of useful information in the form of a feedback report can stimulate an appetite for more information.

### Limitations

Social desirability likely played a role in the survey responses about the usefulness of the feedback reports. Respondents understood that the intervention was about quality improvement—a socially desirable outcome—possibly resulting in overly positive assessments of the usefulness of the feedback report intervention. However, study RAs delivered the surveys (rather than LTC managers or administrators). Participants filled out the survey independently, and were assured their responses were anonymous and would not be shared with colleagues. For these reasons, we do not believe that social desirability had a large influence on responses.

We used a convenience sample, so our study group might be comprised of individuals with a more positive evaluation of the feedback reports than those who did not complete the survey. As a result, perceptions of the report’s usefulness could be more reflective of the opinions of people already eager to receive feedback or other forms of data. The logistic regression findings support this, as those who reported interest in other types of data also found the report information useful for making changes to resident care.

Reliable response rates were difficult to determine because we could not assess what proportion of the total providers working at any point in time participated in a survey. None of the facilities included in the study have a standardized or comprehensive approach to determine the number of providers working at any given point in time. Response rates in each of the final three survey cycles for the pooled sample of all provider groups (from the larger study) ranged from 49.9% to 83.4%, with a mean of 64.4%. We based this on a comparison of the number of surveys distributed with those returned. We did not track which care providers received a survey and, thus, cannot report response rates specific to unregulated care providers, but only for all providers as a whole; only the completed surveys identify the provider status of the respondent.

### Recommendations

One clear recommendation from our study is to include unregulated providers in quality improvement efforts, particularly when they include an informational component such as feedback reports. These providers are an important part of the care team in LTC, and including them in a quality improvement intervention could be a factor in an intervention’s success.

The unregulated care provider traditionally holds a position with little decision-making power. However, unregulated providers may have a substantial amount of informal power generated through the sheer number of them in the system, as well as the access to information they have by being in direct contact with residents on a 24-hour basis [24,40,41]. Managers and other decision-makers in LTC organizations should consider how they can include unregulated care providers in quality improvement efforts to mobilize this informal power. Our findings suggest that unregulated providers not only understand feedback reports related to quality of care, but may also be able to act as change agents if empowered with sufficient self-efficacy. We will explore this issue in future research using self-efficacy questions from the third component of the post-feedback survey.

This analysis supports recommendations for clinical educators who teach unregulated providers in LTC settings. While quality of care may be covered in orientation programs or other in-services, it is not given much time or weight in the current curriculum or continuing education programs for unregulated providers. Additional emphasis on education around quality of care is warranted, given the evidence that these providers can understand the information and find it useful in their practice. However, as we discuss above, simply giving an understandable feedback report to unregulated care providers may not be enough to guarantee practice change in LTC environments. This may be because these environments are characterized by hierarchical decision-making and management structures. Innovative approaches to supporting active engagement of the unregulated provider in quality improvement interventions should be considered.

The lack of a significant relationship between usefulness and understanding in the logistic regression suggests a need to examine the relationship between these variables in more depth. We will explore this relationship in follow-up focus groups with unregulated providers from two of the facilities included in this study. In future work, we will also examine the extent to which resident outcomes changed over the duration of the study and the extent to which this audit and feedback intervention resulted in changes to self-reported intent to assess pain. However, even with the need for further research and analysis in this area, our findings can be used to inform education and curricular development for unregulated and other providers in LTC settings.

## Conclusions

Our findings are exploratory and suggest that unregulated care providers are an appropriate target for feedback reports providing unit-level aggregate resident data. They also indicate the need to explore contextual factors possibly affecting the unregulated care providers’ ability to improve their own care practices, and under what circumstances they feel comfortable making changes. Given that unregulated care providers comprise the largest segment of care providers in LTC settings, further engaging them in knowledge sharing and decision-making is important; ignoring this group of care providers in care planning discussions and decisions would, at best, maintain the status quo. Including and even targeting this group of providers may result in important advances in improving the quality of resident care.

## Abbreviations

LPN: Licensed practical nurse; LTC: Long term care; RA: Research assistant; RAI: Resident assessment instrument; RN: Registered nurse; RPN: Registered psychiatric nurse.

## Competing interests

The authors have no competing interests to declare.

## Authors’ contributions

KDF contributed to the initial conceptualization of the study, oversaw data collection and analysis, and participated in drafting the manuscript. HMO collected data, assisted in the analysis, and participated in drafting the manuscript. AMB contributed to the initial conceptualization of the study and participated in drafting the manuscript. MABB collected data, assisted in the analysis, and participated in drafting the manuscript. AES conceptualized the study and design, oversaw data collection and analysis, and participated in drafting the manuscript. All authors read and approved the final manuscript.

## Pre-publication history

The pre-publication history for this paper can be accessed here:

http://www.biomedcentral.com/1471-2318/13/15/prepub

## Supplementary Material

Additional file 1 **Post-feedback survey. **Description of data: Post-feedback survey used in the Data for Improvement and Clinical Excellence (DICE) study. Click here for file
