# Supplementary material for: Unregulated provider perceptions of audit and feedback reports in long-term care: cross-sectional survey findings from a quality improvement intervention
Source: BMC Geriatr. 2013 Feb 13;13:15. doi: 10.1186/1471-2318-13-15 (PMC3598638; doi:10.1186/1471-2318-13-15)
Supplement: Additional file 1 — Post-feedback survey. Description of data: Post-feedback survey used in the Data for Improvement and Clinical Excellence (DICE) study. [file 1471-2318-13-15-S1.pdf]

Data for Improvement and Clinical Excellence (DICE)

Post-Feedback Report Survey

General Information and Instructions:

BEFORE COMPLETING THIS SURVEY, please make sure you have received a copy of the feedback report that was distributed in your unit last week. If not, ask for a copy from the Research Assistants administering this survey and take a few minutes to have a look at it.

This is an **anonymous** survey. None of the questions in this survey let us know who you are, and we are not asking you to identify yourself.

This survey has three sections:

Section A asks you some questions about yourself - which unit you work on, what your job title is, and how long you have been working in this area. We ask these questions so that we can compare answers among people who work in different jobs and on different units.

Section B asks you about the DICE feedback report that was distributed in your facility a week ago. We ask about how much of the report you read, understood, and how useful you felt it was. We also ask several questions to find out how you think you might use the information in the reports. If you work on or cover more than one unit in your usual work, please respond to the questions in this section based on the reports for all units. Otherwise, please respond to the questions in this section based on the report for the unit you usually work on.

Section C asks questions about whether you plan to change any of the ways you find out if a resident is having pain, which only applies to people who give direct care to residents.

Section A: A few questions about you

1. Which unit do you work on? (Check **ONE** if you usually only work on one unit; if you usually work on several units-- for example, as a recreational therapy assistant-- then check **ALL** that apply)
   _____ a. 1
   _____ b. 2
   _____ c. 3
   _____ d. Other (Please specify): ____

Answer Question 2 only if you usually work on several units

2. In which unit do you work on **MOST OF THE TIME** or the unit you are **MOST FAMILIAR** with? (Check **ONE** answer.)
   _____ a. 1
   _____ b. 2
   _____ c. 3
   _____ d. Other (Please specify): ____

3. What is your position title? (Check **ONE**; if you have more than one position, check the one that you work most often)
   _____ a. Care Manager
   _____ b. Registered Nurse
   _____ c. Licensed Practical Nurse
   _____ d. Health Care Aide/Personal Care Attendant
   _____ e. Social Worker
   _____ f. Physical Therapist/Assistant
   _____ g. Recreational Therapist/Assistant
   _____ h. Occupational Therapist/Assistant
   _____ i. Pharmacist
   _____ j. Dietitian
   _____ k. Other (Please specify): ____

4. How long have you been working in long term care?

   If more than 1 year, how many years? __________ (example: 7 years)
   or
   If less than 1 year, how many months? __________ (example: 11 months)

5. How long have you been working on this unit?

   If more than 1 year, how many years? __________ (example: 2 years)
   or
   If less than 1 year, how many months? __________ (example: 5 months)

Section B: What do you think about the DICE feedback report?

We distributed a report that shows information about residents on your unit based from the RAI-MDS 2.0, the system that is used to collect data about residents in nursing homes every three months.

If you work on or cover more than one unit, please answer these questions based on the feedback reports for all the units you work on or cover.

1. Did you receive the report(s)? (Check **ONE** answer.)

   _____ a. Yes
   _____ b. No (Ask for a copy(s) to look at to complete this survey)

2. For which unit(s) did you receive the report? (Check **ALL** that apply.)

   _____ a. 1
   _____ b. 2
   _____ c. 3
   _____ d. Other (Please specify): ____

3. How much of the report did you read? (Check the letter that reflects how much of the report(s) you've read; check **ONE** answer.)

   _____ a. Less than half
   _____ b. About half
   _____ c. More than half
   _____ d. All of it

4. How well do you feel you understood the information that was in the report(s) about residents on your unit? (Check the letter that fits how well you understood the information; check **ONE** answer.)

   _____ a. Less than half
   _____ b. About half
   _____ c. More than half
   _____ d. All of it

5. How much do you feel that the information in the report accurately reflects the way your unit is? (Check **ONE** answer.)

   _____ a. Does not reflect my unit at all
   _____ b. Partially reflects my unit
   _____ c. Reflects my unit very well

6. How useful did you find the report? (Check **ONE** answer.)

_____ a. Not useful
_____ b. Somewhat useful
_____ c. Useful
_____ d. Very useful

7. Do you think the report showed that your unit is doing well or not? (Check **ONE** answer.) **If you are <u>working on more than one unit</u>, think of the unit you work MOST OF THE TIME or the unit you are MOST FAMILIAR with.**

_____ a. The information mostly showed that my unit is doing better than the other 8 units in the study.

_____ b. The information mostly showed that my unit is not doing as well as the other 8 units in the study.

_____ c. The information mostly showed that my unit is doing about the same as the other 8 units in the study.

_____ d. Can't Tell (Please describe): ____

____

____

8. Did you discuss the report with another staff member, either on your unit, or someone who works somewhere else in the facility? (Check **ONE** answer.)

_____ a. Yes **(*Go to Question 8.1 and 8.2*)**
_____ b. No **(*Go to Question 9*)**

8.1 If **YES** in **Question 8**, what type of staff member did you discussed the report with? (Check **ALL** that apply.)

_____ a. Peer (someone else who does the same type of job as you do)
_____ b. Your direct supervisor
_____ c. A care manager
_____ d. A facility administrator
_____ e. Others (Please specify): ____

8.2 If **YES** in **Question 8**, why did you talk to another staff member? (Check **ALL** that apply.)

_____ i. I wanted to find out what they thought about the report.

_____ ii. I wanted to get their advice about how to make things better for residents based on the report

_____ iii. Other reason (Please tell us what this reason is):

____

____

____

9. Have you discussed previous feedback reports in a staff meeting <u>during the last year</u>? (Check **ONE** answer.)

_____ a. Yes **(*Go to Question 9.1*)**
_____ b. No **(*Go to Question 10*)**

9.1 If **YES** in **Question 9**, how many times? (Check **ONE** answer.)

_____ a. 1-3 times
_____ b. 4-6 times
_____ c. more than 6 times during the last year

10. Does getting this feedback report make you more interested in other types of data (for example, other domains from the MDS-RAI 2.0)? (Check **ONE** answer.)

_____ a. Yes **(*Go to Question 10.1*)**
_____ b. No **(*Go to Question 11*)**

10.1 If **YES** in **Question 10**, what other kinds of information are you interested in?

____

____

____

11. After reading the report, are there other information that would you like to know from the report? (Check **ONE** answer**.)**

_____ a. Yes ***(Go to Question 11.1*)**
_____ b. No **(*Go to Question 12*)**

11.1 If **YES** in **Question 11**, what other information would you like to know from the report that would be MOST helpful to you? (Check **ONE** answer.)

_____ a. Information about the reasons for the information in the report (Why things are the way they are)

_____ b. Information about best practices for specific kinds of care for residents

_____ c. Information about how other units or facilities have addressed similar kinds of concerns

_____ d. Information about whether things are changing over time

_____ e. Other kinds of information (Please describe):

____

____

____

12. Did the report give you information that you could use to make changes in the way you take care of residents? (Check **ONE** answer.)

_____ a. Yes ***(Go to Question 12.1*)**
_____ b. No **(*Go to Section C*)**

12.1 If **YES** in **Question 12,** what changes would you like to make in how you take care of residents? (Check **ALL** that apply.)

____ a. Change the way you assess residents

____ b. Change the way you assist residents in their activities of daily living

____ c. Change the daily schedule for residents

____ d. Change activities available for residents

____ e. Change policies that affect residents or resident care

____ f. Other kinds of change (Please describe): ________________________________

____

____

Section C: Intent to Change Care Practices

Do you provide direct patient care?

____ a. Yes (***Answer Questions 1 to 21 and fill out the GENERAL COMMENTS section on the last page***)

____ b. No (***DO NOT answer Questions 1 to 21; Please write your GENERAL COMMENTS in the box provided on the last page***)

All questions refer to changing how you find out if residents you take care of are having <u>pain</u> (Circle ONE number <u>between 1 and 7</u> for each statement):

1. If I assess resident pain levels, I will feel that I am doing something positive for the resident:

   1 2 3 4 5 6 7
   Very unlikely Very likely

2. It causes a lot of worry and concern for a resident if I assess their pain level:

   1 2 3 4 5 6 7
   Very unlikely Very likely

3. If I assess level of pain, I will detect any problems at an early stage:

1 2 3 4 5 6 7
Very unlikely Very likely

4. If I assess level of pain, I will have to deal with a resident more often:

1 2 3 4 5 6 7
Very unlikely Very likely

5. The methods I use to assess level of pain are not very accurate:

1 2 3 4 5 6 7
Very unlikely Very likely

6. When I am assessing level of pain, I feel rushed:

1 2 3 4 5 6 7
Very unlikely Very likely

7. Having to deal with a resident more often is:

1 2 3 4 5 6 7
Extremely undesirable Extremely desirable

8. Doing something positive for the resident is:

1 2 3 4 5 6 7
Extremely undesirable Extremely desirable

9. Assessing level of pain for each resident early and often is:

1 2 3 4 5 6 7
Extremely undesirable Extremely desirable

10. Doing what other caregivers **like me** in my unit/facility do is important to me (for example, if you are an RN, think about other RNs in your facility):

1 2 3 4 5 6 7
Not at all important — Extremely important

11. Doing what experts in long term care do is important to me:

1 2 3 4 5 6 7
Not at all important — Extremely important

12. The approval of the residents I take care of is important to me:

1 2 3 4 5 6 7
Not at all important — Extremely important

13. People who are important to me think that I should NOT assess level of pain among the residents I care for:

1 2 3 4 5 6 7
Strongly disagree — Strongly agree

14. I expect to assess level of pain in each resident I care for every shift:

1 2 3 4 5 6 7
Strongly disagree — Strongly agree

15. I feel under social pressure to assess residents' level of pain:

1 2 3 4 5 6 7
Strongly disagree — Strongly agree

16. I am confident that I can assess residents' level of pain if I want to:

1 2 3 4 5 6 7
Strongly disagree — Strongly agree

17. Whether I assess residents' level of pain is entirely up to me:

1 2 3 4 5 6 7
Strongly disagree Strongly agree

18. I want to assess residents' level of pain every shift:

1 2 3 4 5 6 7
Strongly disagree Strongly agree

19. It is expected of me that I assess residents' level of pain:

1 2 3 4 5 6 7
Strongly disagree Strongly agree

20. I intend to assess residents' level of pain during each shift:

1 2 3 4 5 6 7
Strongly disagree Strongly agree

21. Out of the next 10 residents you care for, for how many would you expect to assess level of pain? (Circle **ONE** of the numbers **between 0 and 10** below)

0 1 2 3 4 5 6 7 8 9 10

GENERAL COMMENTS:

This is the end of the survey. Thank you.
